# Inserting otorhinolaryngology in the medical course

**DOI:** 10.1016/S1808-8694(15)31011-9

**Published:** 2015-10-19

**Authors:** Regina Helena Garcia Martins

**Affiliations:** Publications Assistant Director

In most medical schools, otorhinolaryngology is inserted in the curriculum in small blocks, with a restrict didactic number of hours, and offered to the students at the 5th or 6th year. The student has a first encounter with the discipline at the end of his/her training, without prior knowledge about otorhinolaryngological themes and details regarding the ENT physical exam. This topic is constantly discussed in the boards of medical schools and one justification they give us is that the major medical disciplines should be given priority in the first years of medical education, so that in the last years a greater theoretical load can better equip the student to understand and, thus, better learn the content offered by specialty disciplines.

These concepts have been changing recently, and in some schools, the Discipline of Otorhinolaryngology has been inserted earlier on in their medical courses, being part of the blocks that emphasize Physiology and Medical Semiology. Having achieved this deserved position in the medical curricula of such institutions has allowed the students to learn the semiology of head and neck from those professionals who better understand the anatomical details of this region of interest, prevalent diseases and the most adequate treatments to be prescribed; thus, the student starts having a global and practical view, and not only theoretical on a certain topic. When we teach otorhinolaryngological semiology and physiology during the medical course, together with the other major medical disciplines, we are able to show the students the real dimensions of our specialty and how vast our working field really is, for such aspect was so far unknown by most of them. Moreover, early on the student starts to perceive how important a multidisciplinary focus is, and to understand the long desired theoretical basis - application program.

Students are not interest in what they do not know. After some years inserted in the courses of Medical Physiology and Semiology, we could notice their greater interest in events of our specialty, in its practice activities, in the medical residency entrance competition and in otorhinolaryngological-based clinical research. One comment we have frequently had from students is that they could not imagine how vast our field of practice is. I believe this to be one of the ways to have our specialty recognized as a major discipline in Medical Teaching Institutions.

